# Effects of sedentary discontinuous intervention on physiological function and mental health of middle school students

**DOI:** 10.3389/fpubh.2025.1662196

**Published:** 2025-09-18

**Authors:** Wanjun Li, Qiang Tang, Xingzhi Yan, Bingcai Yan

**Affiliations:** ^1^School of Educational Science, Xinjiang Normal University, Urumqi, China; ^2^Biological Education Teaching and Research Group, Chongqing Tongnan Experimental Middle School, Chongqing, China; ^3^Teaching Research Department, Institute for Teacher Advancement of Tongnan District, Chongqing, China; ^4^Department of Physical Education Teaching, Qingdao University of Technology, Qingdao, Shandong, China

**Keywords:** middle school students, being sedentary, intermittent intervention, mental health, physical health

## Abstract

**Background:**

To explore the effects of sedentary discontinuity on the physical and mental health of middle school students, and to provide reference for improving the health level of middle school students.

**Methods:**

180 junior high school students were included in this study. The study lasted for one school year. The experimental group added 4 times of 5-8 minutes of physical exercise between classes every day, while the control group arranged the physical exercise between classes independently. Body composition, blood pressure, heart rate variability, diopter and maximum oxygen intake (VO_2_max) were monitored before and after intervention, and the differences of each index between the two groups and before and after intervention were compared.

**Results:**

Before intervention, there were no significant differences in body fat percentage (fat%), muscle mass, systolic blood pressure (SBP), diastolic blood pressure (DBP), VO_2_max, heart rate variability and diopter between 2 groups (*P* > 0.05). After one academic year of exercise intervention, fat%, SBP, DBP and diopter of the experimental group were lower than those of the control group. VO_2_max, skeletal muscle mass, RR interval standard deviation (SDNN), root mean square (RMSSD) of the difference between adjacent RR intervals, low-band area (LF) of the area under HRV curve, high-band area (HF) of the area under HRV curve, TP, and sympathetic/vagal balance index (LF/HF) of the experimental group were all higher than those of the control group (*P* < 0.05). Significant improvements were observed in learning burnout, depression levels, and peer relationships following the intervention.

**Conclusion:**

The results of this study suggest that sedentary intermittent intervention can effectively improve the physical health level of adolescents and reduce the risk of cardiovascular disease. Schools should actively carry out physical exercise during recess to help students grow up healthily.

## Introduction

In recent years, the excessive use of electronic products and the continuous increase of academic pressure have made sedentary behavior become the normal performance of young people’s study and life. Yang Jian et al. (1) found that 86.67 and 63.67% of middle school students in Henan had more than 8 h of sedentary time on school days and rest days, respectively. According to Wang Fubaihui et al. (2), the sedentary time of Chinese teenagers on school days is about 6.5 h, and the sedentary time on rest days is more than 10 h. The Global Physical Activity Report for Children and Adolescents shows that (3) nearly 80% of Chinese children and adolescents exceed the recommended amount of sedentary time. Epidemiological studies have found that sedentary behavior is closely related to lower VO_2_ Max, muscle strength and flexibility levels in adolescents. At the same time, relevant reports have shown that sedentary behavior has a significant dose relationship with cardiovascular diseases such as adolescent obesity (4), hypertension (5), inflammation (6, 7), metabolic syndrome and psychological diseases such as depression. The occurrence of the above chronic diseases caused by long-term sitting may be related to metabolic changes, hemodynamic changes and inflammation. It is well known that sedentary behavior can significantly reduce muscle activity and reduce blood circulation rate, thus increasing the risk of thrombosis. Long-term sedentary behavior will further inhibit muscle metabolism of glucose, which may lead to insulin resistance and diabetes. In addition, long-term sitting will also lead to the increase of inflammatory markers and oxidative stress, all of which have been proved to be the key to the occurrence and development of chronic diseases. How to reduce the sedentary behavior of adolescents and offset the negative effects of sedentary behavior on health has become an urgent public health problem. Healy‘s (8) study revealed for the first time the positive effects of sedentary intermittent behavior on obesity, sugar and lipid metabolism in adults. In recent years, more and more studies have found that sedentary intermittent intervention has a significant impact on the health level of college students (9), office workers (10) and the older adults (11). However, there are few reports on the effects of intermittent sedentary intervention on middle school students.

Based on this, this study takes junior middle school students as the research object, and explores the impact of increasing fragmented physical exercise time on the health level of sedentary adolescents and children by carrying out physical exercise between classes, so as to provide empirical evidence for effectively avoiding the potential health risks brought by sedentary adolescents and children.

## Research object and method

### Research object

The study was conducted in a middle school in Chongqing in September 2021. During the whole experimental period, all subjects were not infected with COVID-19. The COVID-19 epidemic did not affect the normal teaching activities of the school where the experimental subjects were located. Therefore, the results of this experiment are basically not affected by the COVID-19. According to G Power 3.1 software, for a repeated measures analysis of variance (RM-ANOVA) with a significance level of *α* = 0.05 and a medium effect size (*f* = 0.25), the total sample size needed to achieve a statistical power of 0.95 is at least 54 participants. Considering a 10% dropout rate, this study requires at least 60 experimental subjects. This study used stratified random sampling method. Unit by class. Randomly select 9 experimental subjects from each class. A total of 180 people were selected,with an average age of 12.06 ± 0.86(2021), including 74 males and 96 females. Exclusion criteria: (1) patients with cardiovascular and cerebrovascular diseases or other health problems need to take drugs for a long time, (2) Those who are not suitable for physical exercise, (3) The weight has fluctuated greatly in the past 6 months (Over 10 kg). Conduct a full staff questionnaire survey at the school where the experimental subjects are located to collect the above information. Provide reference and basis for the inclusion of eligible subjects. This study was approved by Ethics Committee of Xinjiang Normal University. The research subjects and their guardians fully understand the basic situation of the experiment, fully understand the experimental process and precautions, and sign an informed consent form Pre-test (Table 1).

**Table 1:** Basic information of the experimental subjects.

Group	Sex (female %)	Age
Experimental group	45.6	12.18 ± 1.18
Control group	43.4	11.96 ± 0.36
*p*	0.775	0.87

## Methods

### Intervention methods

The experimental classes were coded, and the subjects were randomly divided into exercise intervention group and blank control group with spss20. The experimental intervention was carried out for one academic year, which was mainly carried out during the 10-min break between classes, and physical exercise activities were carried out four times a day. The intervention content is shown in Table 2. During the experiment, 5 people were injured due to sports, 2 people transferred to other schools and withdrew from the experiment. In the end, a total of 7 people withdrew from the experiment and 173 people completed the intervention. Intervention group (*N* = 83) and control group (*N* = 90).

**Table 2:** Experimental groups and intervention methods.

Group	Components	Experimental intervention
Experimental group	Frequency intensity Content	4 times a day, 5–8 min each time, a total of one school year During the adaptation phase (1 to 4 weeks), HRMAX 60 to 70% moderate intensity aerobic exercise was performed. In the experimental stage (5 ~ the end of the experiment), the HRMAX 80% ~ 85% medium-high intensity aerobic exercise was performed. In the experimental group, cardiopulmonary endurance training and strength training were the main training methods. The content of cardiopulmonary endurance training includes skipping rope, running back and high leg lift, etc. The strength training is mainly based on lunge walking, belly curl, plank support, static squat and push up.
Control group		Students are free to relax after class. Control group students were instructed to engage in their usual break-time activities. Based on school observations and teacher reports, these typically included socializing in the classroom or hallways, light walking, using restrooms, or preparing for the next class. Organized physical activity or structured exercise was not encouraged or provided. As per the school’s standard practice, control group students managed their break times independently without specific intervention. Information on their typical activities was gathered through baseline questionnaires assessing habitual break-time behavior or interviews with school staff describing common practices

### Academic performance

The study evaluated students’ academic performance on the basis of admission tests and final exams for the 2021 academic year.

### Learning burnout

In this study, the adolescent learning Burnout Scale compiled by Wu Yan et al. (12) was used to evaluate the learning burnout level of the experimental subjects, which was composed of 16 items in 3 dimensions (emotional exhaustion, academic alienation and low sense of accomplishment). The 5-level rating system is adopted, and the higher the total score is, the more serious the learning burnout is.

### Peer relationship

In this study, the peer relationship satisfaction scale compiled by Wei Yunhua et al. (13) was used to evaluate the peer relationship quality of the experimental subjects. The scale consisted of 20 items and adopted a 5-level scoring method, scoring 1–5 points from “completely inconsistent” to “completely consistent,” with the total score ranging from 20 to 100 points.

### Depression level

Using popular science research center depression rating scale (Center for Epidemiological Survey Depression Scale, CES - D) (14) to evaluate students’ depression level, the scale is composed of 20 items, use level 4 score system, scored more than 15 points, said there could be a depressive symptoms.

### Blood pressure

The blood pressure of the subjects was measured by an upper arm medical electronic sphygmomanometer. Before the test, the test subjects were required to rest for at least 5 min, and the blood pressure of the right upper arm was measured in a seated position. The blood pressure of the test subjects was measured twice, with an interval of 1-2 min. The average value of the two tests was taken, and the blood pressure of the test subjects was determined by referring to the Chinese Blood Pressure Reference Standards for Age and Height of males and females aged 3–17 years old.

### Physical testing

The test of heart rate variability in subjects using the Madic Heart Rate Variability Analyzer (SA-3000P) requires the subjects to be tested in a quiet, warm session, completely relaxed and quiet during the test, the doctor clips electrodes to the subject’s limbs, the entire test lasts for 5 min, and the data is collected after the test. The body composition of the subject was monitored using a bioelectrical impedance tester (product model: In Body 570). Before the test, the subjects were required to empty their stomachs, bladder, and limit their water intake. During the test, the students wore single clothes and bare feet, stepped on the electrode sheet with their arms extended and held the handle tightly, and recorded the body composition data of the test subjects. Previous studies have confirmed the effectiveness of In Body 570 in children’s body composition testing. Therefore, this study used In Body 570 to evaluate the body composition of the experimental subjects (1, 15).

### Cardiorespiratory endurance

In this study, the cardiopulmonary endurance test was carried out on the subjects in the form of 20-m shuttle Run test. The subjects stood at the starting line and followed the musical instruction to run, and the running speed gradually increased. The initial speed is 8.5 km/h, and for every 1 level increase in music rhythm, the running speed increases by 0.5 km/h. When the subjects failed to reach the line after the musical cue was raised for two consecutive times or they failed to follow the musical rhythm to run and give up the test, the test was over and the number of rewinding times of the subjects were recorded. The formula was used to calculate the maximal aerobic speed, (MAS) and maximal oxygen uptake of the subjects, VO_2_max (ml/kg/min) = 31.025 + 3.238 × MAS-3.248 × age +0.1536 × MAS × age (16).

### Diopter measurement

Diopter measurement was performed using subjective refraction conducted by an experienced optometrist. Participants stood at a 5-meter distance from the visual acuity chart, with one eye occluded while the other eye identified the orientation of internationally standardized ‘E’ optotypes to establish baseline uncorrected visual acuity. Subsequently, trial lens frames were fitted, and monocular refraction was systematically refined by incrementally adjusting corrective lenses in 0.25 diopter (D) steps until full clarity of all optotypes on Line 5.0 of the GB/T 11533-2011 Standard Logarithmic Visual Acuity Chart was achieved for each eye, thereby determining the final prescription.

### Data analysis

Statistical analysis was conducted on all data using SPSS 22.0, and the results were expressed as M ± SD. The data subjected to normality and homogeneity of variance tests were analyzed for inter group differences using independent sample t-tests, intra group differences using paired sample t-tests. The significance level of the difference in data analysis results is set as *p* < 0.01 as very significant, and 0.01 < *p* < 0.05 as significant.

## Results

### Changes in subjects’ academic performance before and after the experiment

As shown in Table 3, in the comparison of the changes of test scores between the two groups, we found that the experimental group had significantly higher increases in math, Chinese and English scores than the control group (*p* = 0.043, 0.011, 0.000).

**Table 3:** Changes in subjects’ academic performance before and after the experiment.

Variable name	Group	M ± SD
Changes in math scores	Control group	2.66 ± 10.24
	Experimental group	5.81 ± 9.06^*^
Changes in language scores	Control group	2.05 ± 7.49
	Experimental group	4.75 ± 6.14^*^
Changes in English scores	Control group	−1.17 ± 9.60
	Experimental group	5.46 ± 10.17^**^

Compared with the control group, * means *p* < 0.05, ** means *p* < 0.01.

### Changes in the mental health level of subjects before and after the experiment

As shown in Table 4, learning burnout in the experimental group decreased significantly before and after the experiment (*p* < 0.05). After the experiment, the comparison of the learning burnout level between the two groups showed that the learning burnout level of the control group was significantly higher than that of the experimental group (*p* < 0.05). The depression level of the experimental group was significantly decreased (*p* < 0.01). After the experiment, the comparison of depression level between the two groups showed that the learning burnout level of the control group was significantly higher than that of the experimental group (*p* < 0.01). The peer relationship quality of the experimental group was significantly improved after the experiment (*p* < 0.01), and the comparison results of peer relationship quality between the two groups showed that the peer relationship quality of the experimental group was significantly higher than that of the control group (*p* < 0.05).

**Table 4:** Changes in the mental health level of the subjects before and after the experiment.

Variable name	Group	Pre-test	Post-test
Learning burnout	Control group	33.63 ± 6.16	34.24 ± 5.32
	Experimental group	32.38 ± 5.58	30.48 ± 2.56^**@^
Depression level	Control group	10.34 ± 4.08	10.08 ± 3.36
	Experimental group	10.13 ± 3.66	7.83 ± 3.66^**@@^
Peer relationship	Control group	57.42 ± 11.42	58.04 ± 14.87
	Experimental group	57.58 ± 10.97	62.12 ± 11.77^**@^

Compared with the control group, * means *p* < 0.05, ** means *p* < 0.01, compared with Pre-test, @ means *p* < 0.05, @@ means *p* < 0.01.

### Changes in the physical health level of subjects before and after the experiment

Intra-group comparison showed that before and after intervention, the systolic blood pressure and diastolic blood pressure of the experimental group had no statistical significance (*p* > 0.05), while the systolic blood pressure and diastolic blood pressure of the control group were both increased compared with before intervention, with statistical significance (*p* < 0.05). The results of inter-group comparison showed that there was no significant difference in blood pressure between the two groups Pre-test, and the diastolic blood pressure and systolic blood pressure of the control group were significantly higher than those of the experimental group after the experiment (*p* < 0.05).

Intra-group comparison showed that VO_2_max increased in both experimental group and control group before and after intervention, and the differences were statistically significant (*p* < 0.05). Inter-group comparison showed that after intervention, the VO_2_max of the experimental group was higher than that of the control group, and the differences between the groups were statistically significant (*p* < 0.05).

Intra-group comparison showed that before and after intervention, body fat percentage decreased and muscle mass increased in the experimental group, with statistical significance (*p* < 0.05), while body fat percentage and muscle mass increased in the control group, with statistical significance (*p* < 0.05). Inter-group comparison showed that the experimental group had lower body fat percentage and higher muscle mass after intervention. The differences were statistically significant (*p* < 0.05).

Intra-group comparison showed that SDNN (ms), RMSSD (ms), LFn.u., HFn.u., TP (ms2) of the experimental group were all increased before and after intervention, and LF/HF were decreased, with statistical significance (*p* < 0.05). In control group, SDNN (ms), LFn.u., LF/HF were increased, and the differences were statistically significant (*p* < 0.05), while the other heart rate variability indexes were not statistically significant (*p* > 0.05).

Inter-group comparison showed that SDNN (ms), RMSSD (ms), LFn.u., HFn.u., TP (ms2) were higher in the experimental group after intervention, and LF/HF was lower, with statistical significance (*p* < 0.05).

Intra-group comparison showed that the diopter of the experimental group and the control group decreased before and after intervention, and the difference was statistically significant (*p* < 0.05). Intra-group comparison showed that the diopter of the experimental group was lower after intervention, and the difference was statistically significant (*p* < 0.05) (see Table 5).

**Table 5:** Cardiopulmonary function before and after the experiment.

Variable name	Group		Pre-test	Post-test
Blood pressure	Control group	DBP	66.67 ± 5.07	69.53 ± 7.38^@@^
		SBP	105.10 ± 9.14	109.20 ± 11.79^@^
	Experimental group	DBP	65.93 ± 6.50	66.63 ± 5.64^**^
		SBP	104.97 ± 8.84	105.48 ± 10.24^*^
Maximal oxygen uptake	Control group		44.39 ± 3.12	46.20 ± 3.08^@^
	Experimental group		45.00 ± 3.39	49.31 ± 3.41^**@@^
Body fat percentage	Control group		22.13 ± 7.60	24.39 ± 8.65^@@^
	Experimental group		23.26 ± 10.01	21.93 ± 7.14^*@^
Muscle mass	Control group		35.80 ± 4.33	35.25 ± 4.30
	Experimental group		35.67 ± 4.02	37.31 ± 4.83*^@@^
SDNN (ms)	Control group		51.63 ± 11.83	54.61 ± 12.06^@^
	Experimental group		51.81 ± 13.75	58.71 ± 13.76*^@@^
RMSSD (ms)	Control group		49.76 ± 10.55	48.49 ± 11.47
	Experimental group		49.26 ± 14.00	52.72 ± 14.58*^@@^
LFn.u.	Control group		49.09 ± 12.11	52.50 ± 14.49^@@^
	Experimental group		48.11 ± 12.77	56.88 ± 13.79*^@@^
HFn.u.	Control group		53.92 ± 7.69	53.66 ± 7.91
	Experimental group		54.43 ± 8.39	56.39 ± 8.39*^@^
TP (ms^2^)	Control group		7.41 ± 0.71	7.49 ± 0.9
	Experimental group		7.47 ± 0.84	7.78 ± 0.8*^@^
LF/HF	Control group		1.27 ± 0.44	1.57 ± 0.47^@@^
	Experimental group		1.35 ± 0.36	0.65 ± 0.32**^@@^
Diopter	Control group		−83.46.40 ± 105.09	−126.63 ± 123.38^@@^
	Experimental group		−71.14 ± 98.48	−91.31 ± 105.26^**@@^

Compared with the control group, * means *p* < 0.05, ** means *p* < 0.01, compared with Pre-test, @ means *p* < 0.05, @@ means *p* < 0.01. Diopter values: Negative sign (−) denotes myopia.

## Discussion

With the change of production and life style, the popularization of modern intelligent tools, the necessary physical activity of human beings continues to decrease, and sedentary life has become the norm of people’s work and life. Studies have shown that sedentary behavior in adolescents is one of the risk factors for physical diseases such as overweight, obesity, hypertension, cardiometabolic risk (17) and psychological disorders such as depression, anxiety (18) and suicide attempt (18). How to reduce the sedentary time of adolescents and alleviate or eliminate the negative impact of sedentary time on the physical and mental health of adolescents has become a worldwide public health problem that needs to be solved urgently. However, the close curriculum arrangement of the school day makes it difficult to reduce the sedentary time of adolescents, so it is more worthwhile to explore and study the potential risks brought by different ways to curb sedentary time.

This study found that recess physical exercise can significantly improve junior middle school students’ academic performance and mental health level, muscle mass and maximal oxygen uptake, delay the increase of blood pressure and the decrease of vision, reduce their body fat percentage, and improve their heart rate variability.

Gonzalez-Sicilia (19), PindusDM (20) and Isensee et al. (21) found in their studies that increasing physical exercise has a positive effect on improving students’ academic performance. Although the significant benefits of increased physical activity in improving academic performance have been widely recognized (22–24), these studies have focused more on the effects of after-school and longer periods of physical activity on academic performance, and relatively little research has been done on the relationship between recess physical activity and academic performance. However, limited studies have also shown that increasing pre-class exercise can significantly improve children’s cognitive function and thus positively influence academic performance (25).

While our results demonstrate a significant positive association between recess physical activity and academic performance, the underlying neurobiological mechanisms require further investigation. Potential pathways suggested by prior research include increased secretion of brain-derived neurotrophic factor (BDNF) (26, 27) and enhanced neurovascular adaptation with synaptic plasticity (28, 29), which may collectively improve cognitive functions—particularly attention and working memory—that support academic engagement (30). Critically, as BDNF levels and neurostructural changes were not quantified in the present study, these remain hypothetical pathways warranting verification through future biomarker-integrated trials employing serum assays and neuroimaging techniques.

In addition, studies have pointed out that there is a significant increase in sedentary time among adolescents from primary school to middle school, and epidemiological studies have shown that continuous sedentary time will increase the risk of overweight and obesity, cognitive function decline, and depressive symptoms in adolescents and children (31–34), which will adversely affect their academic performance. Increasing physical activity between classes significantly reduced the amount of time students spent sitting and improved their academic performance.

Continuous sedentary state will lead to the imbalance of energy consumption and intake of the body, and sedentary state will reduce the oxidation of lipids in muscle tissue, which will lead to continuous accumulation of lipids and eventually lead to overweight and obesity (35, 36). Animal experiments also found that the restriction of activity would lead to the disturbance of the secretion of lipocalin2 (LCN2), which is related to appetite regulation and energy metabolism, and osteocalcin (OCN), which is related to glucose uptake in skeletal muscle, resulting in food intake level and weight gain. On the one hand, the improvement of sedentary intermittent exercise on body composition comes from the increase in energy consumption during and after exercise, which causes the decomposition of body fat and inhibits the accumulation of fat cells. At the same time, exercise stimulates the secretion of leptin, insulin and other hormones and mediates the uptake of blood sugar and blood lipids by skeletal muscle, thus reducing the risk of obesity (37). As early as 2008, Healy et al. found that frequent intermittent sedentary behavior can significantly reduce adult triglycerides and postprandial blood sugar and other indicators (8). Myashia et al. pointed out that obese young people who accumulated 10 times of medium-high intensity intersong exercise for 30 min would have a 39% decrease in TG 7 h after meals the next day (38). Bhammar et al. showed that a 2-min interval of moderate to high intensity every 20 min significantly reduced average blood glucose and systolic blood pressure levels in sedentary obese men. All the above studies have shown that sedentary intermittent intervention can have a positive impact on human glucose and lipid metabolism. However, most of the previous studies focused on the older adults, adults and college students and other groups, and this study proves that sedentary intermittent exercise also has potential benefits in young people.

The effect of sedentary intermittent exercise on blood pressure and heart rate variability may be mainly due to the fact that continuous sedentary immobility will lead to the accumulation of lower limb veins, the decrease of arterial blood flow, velocity and shear stress, resulting in excessive release of endothelin 1 and the decrease of bioavailability of nitric oxide (itricoxide NO), which will eventually lead to the damage of vascular endothelial cells, relaxation and relaxation of blood vessels. Formation of hypertension and blood clots (39, 40). Intermittent exercise can enhance the pumping function of lower limbs, promote blood circulation, increase artery dilation, stimulate NO production and availability of endothelial cells, enhance endothelial cell function and improve blood pressure. Heart rate variability is one of the important biomarkers used to assess autonomic nervous system function (ANS), and many studies have shown that higher heart rate variability values, i.e., strong vagal response, are associated with positive health outcomes, while lower heart rate variability indicates inadequate adaptation of the autonomic nervous system and higher sympathetic nerve activity. This study found that sedentary intermittent intervention could significantly improve the heart rate variability of adolescents, and the overall level of HRV and vagal nerve activity in the experimental group were significantly better than those in the control group after intervention. Magnetic resonance imaging shows that prolonged sitting causes significant changes in the activities of the prefrontal cortex, insula and limbic system cortex, and inactivation of the ventromedial prefrontal cortex leads to overactivity of the amygdala, amplifies the perception of fear and pressure, and keeps people in a state of stress with sympathetic excitation and reduced parasympathetic output. In turn, the sympathetic nerve tension such as heart rate, blood pressure and HRV led to changes in cardiovascular activity.

The study found that with the increase of age, the eye diopter of children showed a downward trend. Although intermittent exercise intervention could not completely prevent the decline of children’s diopter, it could effectively slow down the decline rate and delay the occurrence of myopia in children. This is similar to the results of Guggenheim et al. (41). Studies have shown that the benefits of sports in the prevention and treatment of myopia are mainly attributed to the improvement of ocular muscle function and blood circulation during exercise, the decrease of intraocular pressure after exercise, the improvement of choroidal blood flow speed, and the adequate supply of retinal blood, and the higher level of retinal blood supply can effectively promote the development of ocular neuromuscles in children (42). In addition, the reason why this intervention can delay the diopter decline of the experimental subjects may be related to outdoor light. The prevention and treatment mechanism of light on myopia is mainly attributed to the “light-dopamine” hypothesis. Dopamine, as an important neurotransmitter, can effectively inhibit the increase of the length of the eye axis. It was observed in animal experiments that the rate of dopamine release from the retina of chicks increased log-linearly with the increase of light intensity (43). The exercise in this experiment was carried out outdoors, which may regulate the secretion of retinal dopamine by increasing outdoor light (44), and then affect the length of the axis of the eye, so as to prevent myopia.

## Research limitation and future directions

This study demonstrates the efficacy of sedentary discontinuous interventions in improving cardiometabolic and psychosocial outcomes among adolescents, yet several limitations warrant attention. The single-center sample from Chongqing, China constrains geographical and cultural generalizability, necessitating future multi-center trials across diverse educational systems (e.g., Eastern vs. Western curricula) to verify transferability—particularly regarding curriculum-driven activity patterns and social acceptability of interventions. Furthermore, the lack of objective control group monitoring (e.g., accelerometry) impedes definitive causal attribution, we thus strongly endorse implementing wearable activity trackers for quantifying break-time behaviors and video-based behavioral coding for activity typology analysis. The limited sample size (*N* = 120) also precluded robust subgroup analyses, warranting larger cohorts (>500 participants) to investigate dose-response relationships by BMI percentile and moderating effects of socioeconomic status. To address these gaps, we propose a concrete research agenda: Phase 1 (2024–2025) conducts replication studies across three Chinese provinces, Phase 2 (2026–2027) implements cross-cultural RCTs comparing Confucian-heritage and Western schools, Phase 3 integrates interventions into national physical education frameworks for policy-effectiveness trials.

## Conclusion

Through this study, we found that one academic year of recess physical exercise can significantly improve students’ academic performance, alleviate students’ learning burnout and depression, improve the quality of students’ peer relationships, and promote the establishment of students’ interpersonal relationships; In addition, studies have also shown that increasing the time of physical exercise between classes can reduce students’ body fat percentage, increase body muscle mass, delay vision loss and blood pressure increase, increase students’ VO2 Max, and improve their heart rate variability. The results of this experiment suggest that in the context of the continuous increase of students’ learning pressure and learning time, fragmented physical exercise between classes can also relieve students’ psychological pressure, improve students’ physical health level, and promote students’ healthy growth.

## Data Availability

The original contributions presented in the study are included in the article/supplementary material, further inquiries can be directed to the corresponding authors.
